# Taxonomic review of the Asian *Trogloneta* species (Araneae, Mysmenidae)

**DOI:** 10.3897/zookeys.817.30468

**Published:** 2019-01-15

**Authors:** Ya Li, Yucheng Lin

**Affiliations:** 1 Key Laboratory of Bio-resources and Eco-environment (Ministry of Education), College of Life Sciences, Sichuan University, Chengdu, Sichuan 610064, China Sichuan University Chengdu China

**Keywords:** China, identification key, Japan, mysmenids, new combination, new synonymy, taxonomy

## Abstract

Five *Trogloneta* species from Southwest China and Japan are reviewed that two new combinations and a new synonymy are proposed in the current paper: *T.nojimai* (Ono, 2010), **comb. n.** is transferred from *Mysmena*, *T.yunnanense* (Song & Zhu, 1994), **comb. n.** (= *T.denticocleari* Lin & Li, 2008, **syn. n.**) is transferred from *Pholcomma* of the Theridiidae, *T.speciosum* Lin & Li, 2008, *T.uncata* Lin & Li, 2013, and *T.yuensis* Lin & Li, 2013. The female of *T.yuensis* is described for the first time. An identification key and diagnoses are provided for these species, as well as new photographs or illustrations of the genital organs and habitus of *T.yuensis* and *T.yunnanense*.

## Introduction

The genus *Trogloneta* Simon, 1922 (= *Troglonata*, lapsus calami) was erected by Simon (1922) on the basis of an interesting cave spider from France which originally thought to be a member of the *Mysmeneae* group, and placed in the family Theridiidae Sundevall, 1833. It was later transferred to Symphytognathidae Hickman, 1931 by Gertsch (1960), and placed in Mysmenidae Petrunkevitch, 1928 by Forster and Platnick (1977). Brescovit and Lopardo (2008) reviewed a taxonomic history and proposed a syncretic diagnostic for *Trogloneta*. Recent phylogenetic study indicates that *Trogloneta* is sister to the clade comprising *Maymena* Gertsch, 1960 plus Mysmenopsinae (Lopardo and Hormiga 2015), and a consistent diagnosis is also presented for this genus.

Currently, *Trogloneta* consists of eleven described species (World Spider Catalog 2018), and these members are mainly distributed in Europe, China, Brazil, USA, Madeira, and Canary Island, and prefer to living in cryptic microhabitats such as deciduous layer (e.g., *T.cantareira*, *T.cariacica*, and *T.mourai* in Brescovit and Lopardo 2008), or forest canopy (e.g., *T.speciosum* in Lin and Li 2008) or even in dark caves (e.g., *T.granulum* in Simon 1922, “*T.denticocleari*” and *T.uncata* in Lin and Li 2008, 2013b).

In this paper we provide a brief revision of *Trogloneta* species from Asia. The female of *T.yuensis* Lin & Li, 2013 is described for the first time. An identification key is also provided for these Asian *Trogloneta* species.

## Materials and methods

Specimens were collected by hand and preserved in 95% ethanol. They were examined using a Leica M205 C stereomicroscope. Further details were studied under an Olympus BX43 compound microscope. The epigynes were removed and treated with lactic acid before being photographed. Photographs were taken with a Canon EOS 60D wide zoom digital camera (8.5 megapixels) mounted on an Olympus BX 43 compound microscope. The images were montaged using Helicon Focus 3.10 (Khmelik et al. 2006) image stacking software.

All measurements are in millimetres. Leg measurements are given as follow: total length (femur, patella, tibia, metatarsus, and tarsus). Abbreviations in figures or text are as follows:

**AA** apical apophysis

**Acc** accessory gland

**AL** apical lobe

**ALE** anterior lateral eyes

**AME** anterior median eyes

**At** atrium

**CD** copulatory ducts

**CO** copulatory opening

**Co** conductor

**Cy** cymbium

**CyC** cymbial conductor

**CyF** cymbial fold

**CyFs** setae on cymbial fold

**CyP** cymbial process

**E** embolus

**Et** embolic tip

**FD** fertilisation ducts

**Pa** patella

**PC** paracymbium

**PLE** posterior lateral eyes

**PME** posterior median eyes

**S** spermathecae

**SD** spermatic duct

**Sp** scape

**St** subtegulum

**T** tegulum

**TA** tegular apophysis

**Ti** tibia

Abbreviations of specimen depository institutions:

**IZCAS** Institute of Zoology, Chinese Academy of Sciences in Beijing, China

**NHMSU** Natural History Museum of Sichuan University in Chengdu, China

**NSMT** Department of Zoology, National Museum of Nature and Science in Tokyo, Japan

## Taxonomy

### Trogloneta

Taxon classificationAnimaliaAraneaeMysmenidae

Simon, 1922

#### Type species.

*Troglonetagranulum* Simon, 1922.

#### Diagnosis.

*Trogloneta* differs from other mysmenid genera by the following combination of features: AME (or absent) smaller than ALE, all eyes gathered at apex of carapace; one femoral spot on leg I on both sexes (none on leg II); carapace height dimorphism (male carapace higher than female); anterior booklungs reduced; males with shorter, but stout and straight setae comprising the tarsal prolateral row on leg I; male palp huge (at least as big as half carapace), embolus tubular, its tip simple; cymbium with intricate decorations, and cymbial terminal acting as conductor; tegulum broad, usually having a apophysis; epigynal area elevated ventrally, with a scape, accessory glands on valve, and smooth uniform proximal copulatory ducts of increased diameter (Brescovit and Lopardo 2008, Lopardo and Hormiga 2015).

#### Composition.

*T.canariensis* Wunderlich, 1987, *T.cantareira* Brescovit & Lopardo, 2008, *T.cariacica* Brescovit & Lopardo, 2008, *T.granulum* Simon, 1922, *T.madeirensis* Wunderlich, 1987, *T.mourai* Brescovit & Lopardo, 2008, *T.nojimai* (Ono, 2010), comb. n., *T.paradoxa* Gertsch, 1960, *T.speciosum* Lin & Li, 2008, *T.uncata* Lin & Li, 2013, *T.yuensis* Lin & Li, 2013, *T.yunnanense* (Song & Zhu, 1994), comb. n. (= *T.denticocleari* Lin & Li, 2008, syn. n.).

#### Distribution.

China (Chongqing, Guizhou, Hunan, Yunnan), Japan (Honshu), Europe (Austria, Czech Republic, France, Germany, Italy, Poland, Slovakia), USA (Utah, Oregon, California), Brazil (Minas Gerais, Rio de Janeiro, San Paulo, Santa Catarina, Espirito Santo, Parana), Canary Island, Madeira.

##### Key to Asian species of *Trogloneta* Simon, 1922

**Table:** 

1	Abdomen subglobose (Figs 1A, B, 4A–C, 9A–D)	**2**
–	Abdomen pointed dorsally or posteriorly (Figs 2A, B, F, G, 6A, B, E, F)	**6**
2	Males	**3**
–	Females	**5**
3	Embolus stubby, distal tip falcate (Fig. 5A–D)	*** T. uncata ***
–	Embolus long, distal tip spiculate (Figs 1E, 10B, D)	**4**
4	Cymbium strongly modified, with a huge cymbial process (Fig. 10A, C, D)	*** T. yunnanense ***
–	Cymbium moderately modified, cymbial process absent (Fig. 1E, F)	*** T. nojimai ***
5	Epigyne with a short scape, spermathecae globular (Fig. 11C, D)	*** T. yunnanense ***
–	Epigyne with a long scape, spermathecae oviform (Fig. 1G, H)	*** T. nojimai ***
6	AME absent, embolus straight distally and epigynal scape short (Figs 2A, F, 3C–G)	*** T. speciosum ***
–	AME present, embolus hooked distally and epigynal scape long (Figs 6A, B, 7B, 8D)	*** T. yuensis ***

### Trogloneta nojimai

Taxon classificationAnimaliaAraneaeMysmenidae

(Ono, 2010) comb. n.

**Figure 1.**
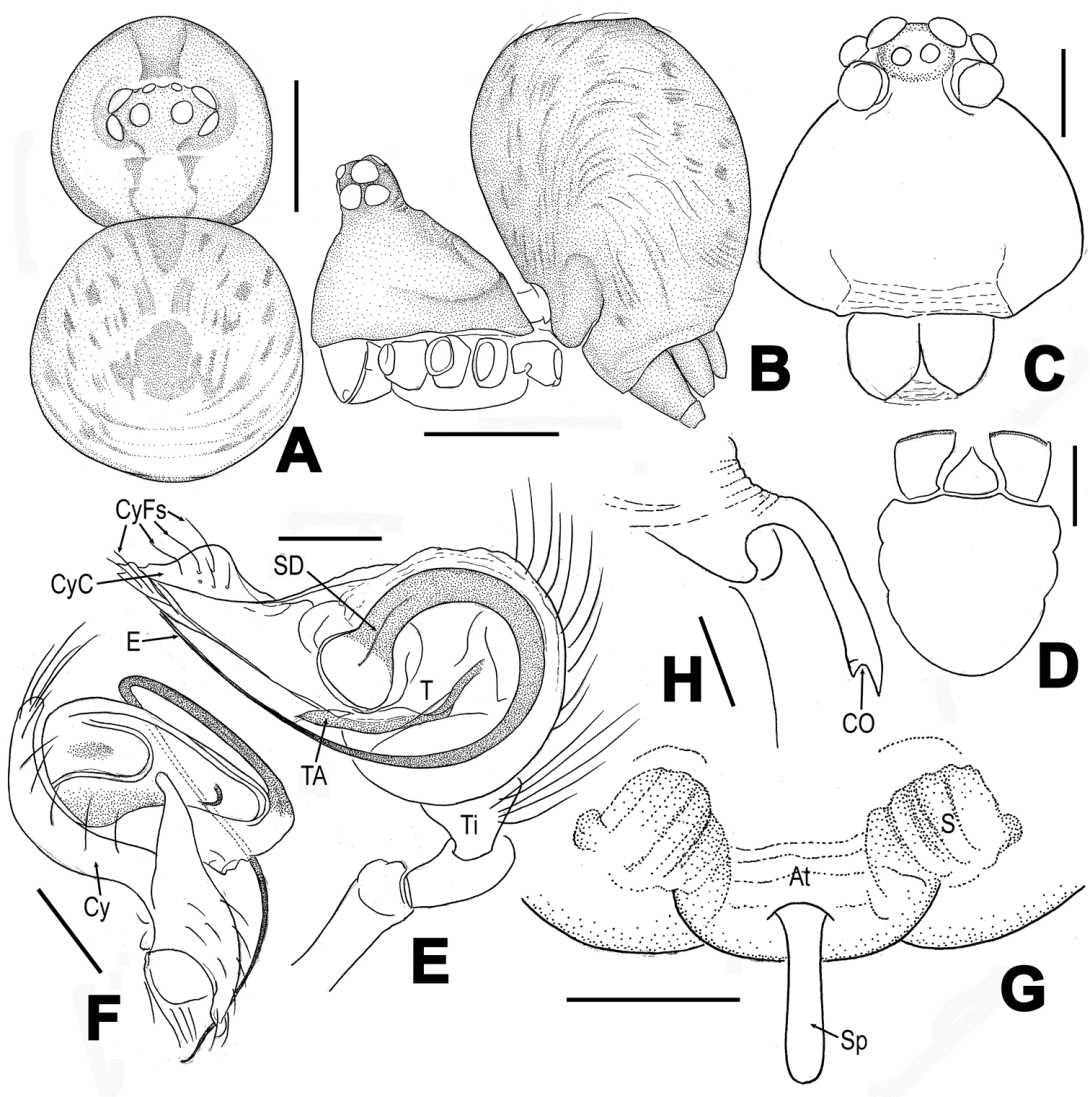
*Troglonetanojimai* (Ono, 2010), comb. n., male holotype (**A–F**) and female paratype (**G, H**) (cited from Ono, 2010, slightly modified). **A, B** habitus (appendages omitted) **C** Prosoma **D** Endites, labium and sternum **E, F** Left palp **G, H** Epigyne **A, F** dorsal **B, H** lateral **C** anterior **D, G** ventral **E** retrolateral. Abbreviations: At atrium; CO copulatory opening; Cy cymbium; CyC cymbial conductor; CyFs setae on cymbial fold; E embolus; S spermathecae, SD spermatic duct; Sp scape; T tegulum, TA tegular apophysis; Ti tibia. Scale bars: 0.25 mm (**A, B)**; 0.10 mm (**C–H)**.

Figure 1 (as modified from Ono, 2010)

Mysmena nojimai Ono, 2010: 2, figs 1–8.

#### Type material.

*Holotype*: ♂ (NSMT-Ar 8515) from JAPAN: Honshu, Okayama Prefecture, Tsuyama-shi, Kamo-cho, Uno, alt. ca. 100 m, 12.VI.2009, K. Nojima leg. *Paratypes*: 1♀ (NSMT-Ar 8516) from JAPAN: Honshu, Okayama Prefecture, Okayama-shi, Kita-ku, Awai, alt. 100–200 m, 6.VI.2009, K. Nojima leg.; 2♀ (NSMT-Ar 8568) from JAPAN: Honshu, Aichi Prefecture, Toyota-shi, Sakaue-cho, Mt. Rokusho-san, alt. ca. 400 m, 18.X.2009, K. Ogata leg. Not examined.

#### Diagnosis.

*Troglonetanojimai* can be distinguished from other congeners but except of *T.yuensis* by the globular abdomen in both sexes (Figure 1A, B), the palp with a long embolus and a distinctly extended cymbial conductor (Figure 1E), and a long and narrow scape in the epigyne (Figure 1G, H). It seems more similar to *T.yuensis* in share with the structure of palp and the configuration of epigyne, but differs from the latter by the abdomen without a dorsal-posteriorly pointed tubercle in both sexes (Figure 1A, B vs. Figure 6A, B, E, F), a straight embolic end and lack of cymbial process (Figure 1E, F vs. Figure 7A–D), and by the oval spermathecae (Figure 1G vs. Figure 8C, D).

#### Taxonomic justification.

Although the type material of this species has not been examined for this study, the shape of palpal bulb, the configuration of epigyne, the patterns of eyes arrangement, and the distinctly elevated, conical carapace in male leave little doubt that it should be a member of the genus *Trogloneta*, but not *Mysmena*. The original illustrations of palp and epigyne of *T.nojimai* by (Ono 2010: figs 1–8) are rather simple and show many important similarities in comparison with those of *T.yuensis* (Figs 7A–D, 8A–D), as one of *Trogloneta* species. Therefore, we propose it as a new combination, *Troglonetanojimai* (Ono, 2010), comb. n., transferring it from *Mysmena*.

#### Distribution.

Japan (Honshu).

### Trogloneta speciosum

Taxon classificationAnimaliaAraneaeMysmenidae

Lin & Li, 2008

Figures 2 , 3

Trogloneta speciosum Lin & Li, 2008: 514, figs 18A–E, 19A–I.

#### Type material.

*Holotype*: ♂ (IZCAS), *paratypes*: 1♂, 3♀ (IZCAS) from CHINA: Yunnan Province, Xishuangbanna Dai Autonomous Prefecture, Mengla County, Menglun Nature Reserve, Primary tropical seasonal rainforest (21°57.420'N, 101°13.020'E; alt. 744±15 m), 30.VII.2007, G. Zheng leg. Examined.

#### Diagnosis.

**Figure 2.**
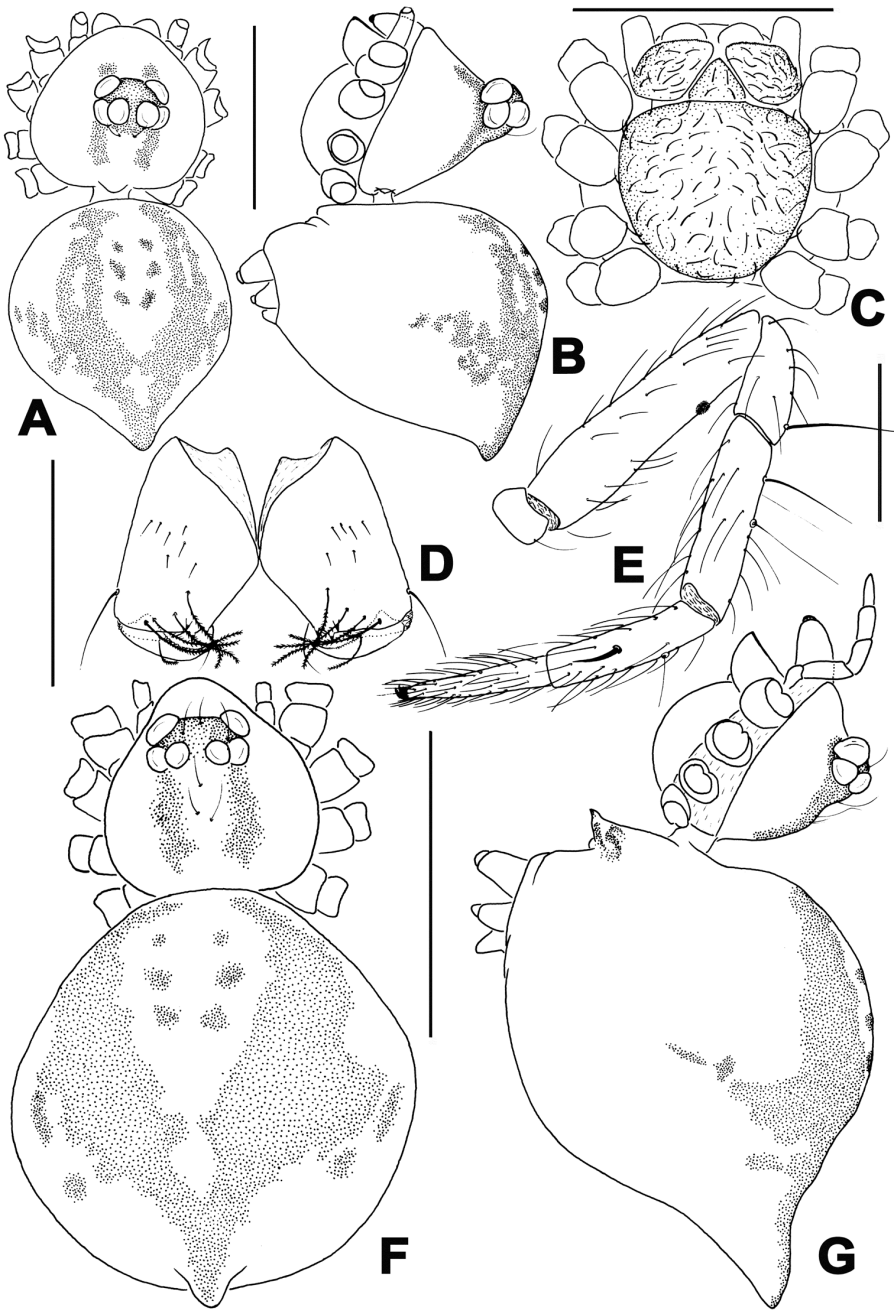
*Troglonetaspeciosum* Lin & Li, 2008, male (**A–E**) and female (**F–G**). **A, B** Habitus **C** Prosoma (appendages omitted) **D** Chelicerae **E** Leg I **F, G** Habitus. **A, F** dorsal **B, G** lateral **C** ventral **D** posterior **E** prolateral. Scale bars: 0.50 mm (**A–D, F, G)**; 0.20 mm (**E)**.

This species differs from all species of *Trogloneta* by the absence of anterior median eyes and the posteriorly pointed abdomen in both sexes (Figure 2A, B, F, G).

**Figure 3.**
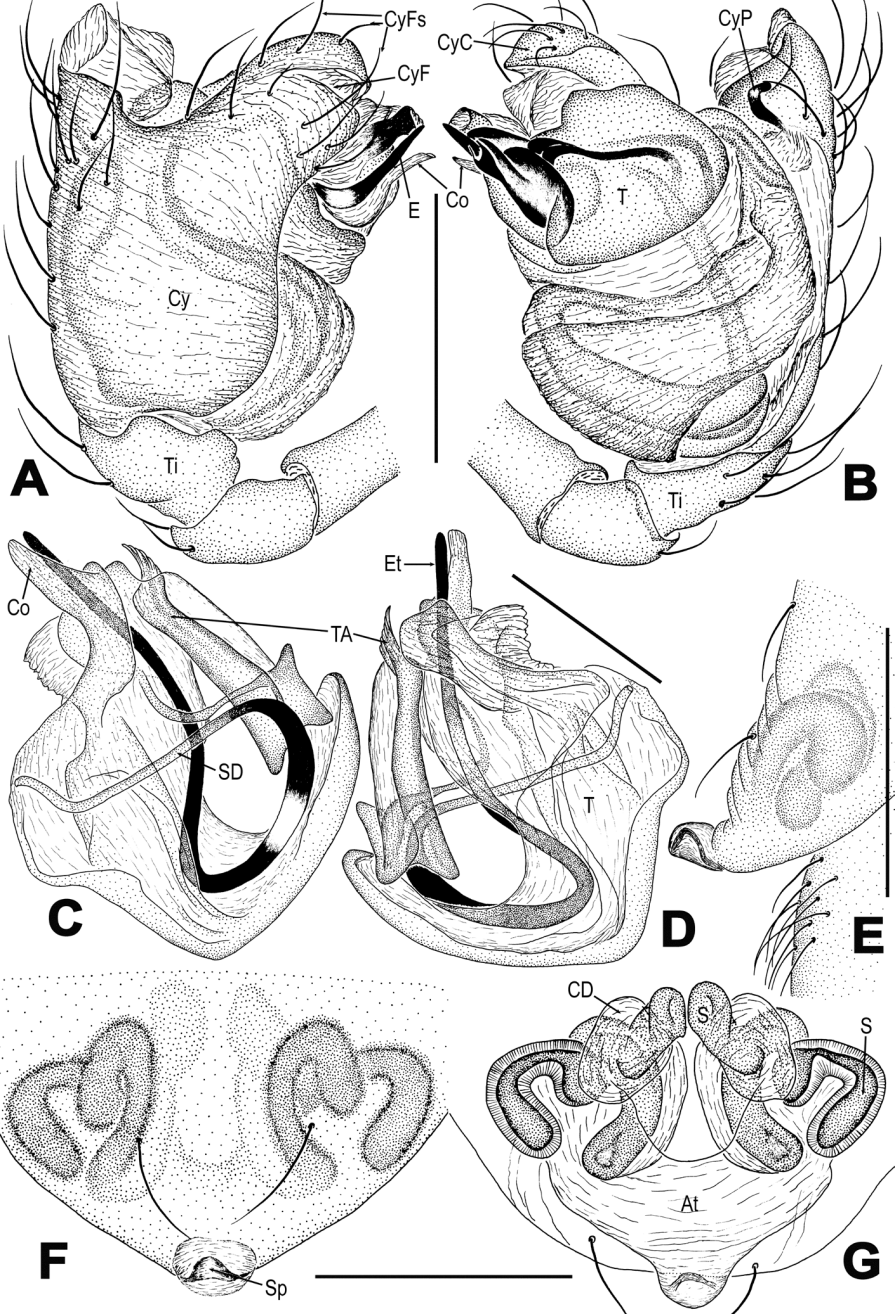
*Troglonetaspeciosum* Lin & Li, 2008, male (**A–D**) and female (**E–G**). **A, B** Left palp **C, D** Embolic division **E, F** Epigyne **G** Vulva. **A** prolateral **B** retrolateral **C, G** dorsal **D, F** ventral **E** lateral. Abbreviations: At atrium; CD copulatory ducts; CO copulatory opening; Co conductor; Cy cymbium; CyC cymbial conductor; CyF cymbial fold; CyFs setae on cymbial fold; CyP cymbial process; E embolus; Et embolic tip; S spermathecae, SD spermatic duct; Sp scape; T tegulum, TA tegular apophysis; Ti tibia. Scale bars: 0.20 mm (**A, B, E–G)**; 0.10 mm (**C, D)**.

#### Remarks.

This is the only species of *Trogloneta* spider ever found living in the tropical rainforest canopy, and is also a relatively rare six-eyed mysmenid species. We have tried to collect it again in the type locality, hoping to obtain some samples for molecular study but unfortunately, more material was not found.

#### Distribution.

China (Yunnan).

### Trogloneta uncata

Taxon classificationAnimaliaAraneaeMysmenidae

Lin & Li, 2013

Figures 4 , 5

Trogloneta uncata Lin & Li, 2013b: 476, figs 23A–G, 24A–D, 25A–D.

#### Type material.

Holotype ♂ (IZCAS) from CHINA: Yunnan Province, Nanjian County, Xiaowan Town, Huilong Mt., Banpoyan Cave (24°56.012'N, 100°18.866'E; alt. 1990 m), 25.VI.2010, C. Wang, Q. Zhao and L. Lin leg. Examined.

#### Diagnosis.

*Troglonetauncata* can be distinguished from Brazilian *Trogloneta* spp. (Brescovit and Lopardo 2008) by the globular abdomen, lack of pointed tubercle posteriorly; from the American *T.paradoxa* (see Gertsch 1960: figs 12, 16) and the Chinese *T.speciosum* (Figure 3A, B), *T.yuensis* (Figure 7A, B), and *T.yunnanense* (Figure 10A, B) by the presence of a spur-shaped cymbial process, and the stout embolus with a falcate tip (Figure 5A–D); from the European *T.canariensis*, the type species *T.granulum*, and *T.madeirensis* from Madeira Is. (see Wunderlich 1987: figs 371, 376, 383) by a stout, hooked embolus and having a cymbial process in the palpal bulb (Figure 5B–D). Female unknown.

**Figure 4.**
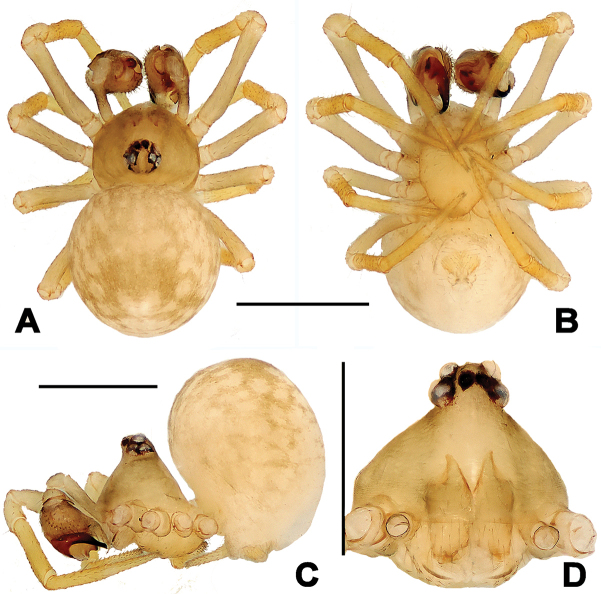
*Troglonetauncata* Lin & Li, 2013, male (**A–D**). **A–C** Habitus **D** Prosoma (appendages omitted) **A** dorsal **B** ventral **C** lateral **D** anterior. Scale bars: 0.50 mm.

**Figure 5.**
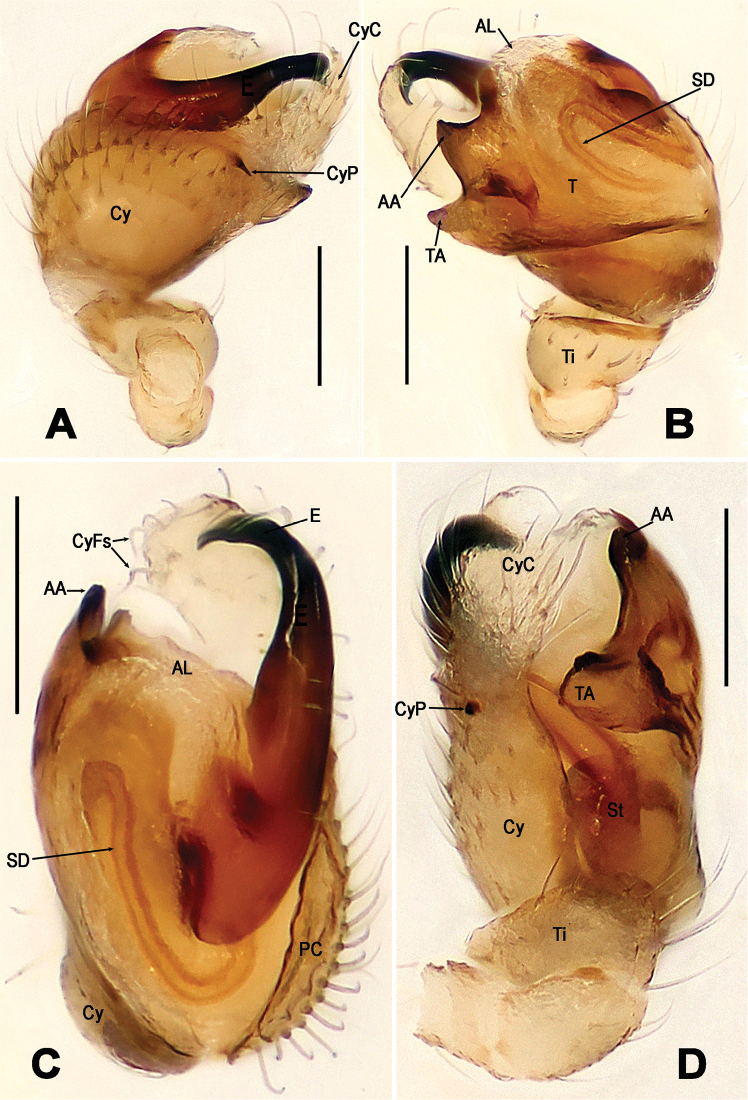
*Troglonetauncata* Lin & Li, 2013, male left pale (**A–D**). **A** prolateral **B** retrolateral **C** apical **D** ventral. Abbreviations: AA apical apophysis; AL apical lobe; Cy cymbium; CyC cymbial conductor; CyFs setae on cymbial fold; CyP cymbial process; E embolus; TA tegular apophysis; PC paracymbium; SD spermatic duct; St subtegulum; T tegulum; Ti tibia. Scale bars: 0.10 mm.

#### Distribution.

China (Yunnan).

### Trogloneta yuensis

Taxon classificationAnimaliaAraneaeMysmenidae

Lin & Li, 2013

Figures 6 , 7 , 8

Trogloneta yuensis Lin & Li, 2013a: 43, figs 8A–E, 9A–B, 10A–F, 11A–B, 12A–E.

#### Type material.

Holotype ♂ (NHMSU) from CHINA: Chongqing City, Beibei District, Jinyun Mt., Guankou (29°50.261'N, 106°23.811'E; alt. 531 m), 5-IV-2010, by sieving, Z. Zhang leg. Examined.

#### Other material.

5♂, 6♀ (NHMSU) from CHINA: Hunan Province, Changsha City, Yuelu District, Yuelu Mt. Parkland, (112°56.526'E, 28°11.211'N; alt. 163 m), 19-IV-2018, by sieving, G. Zhou leg.

#### Diagnosis.

**Figure 6.**
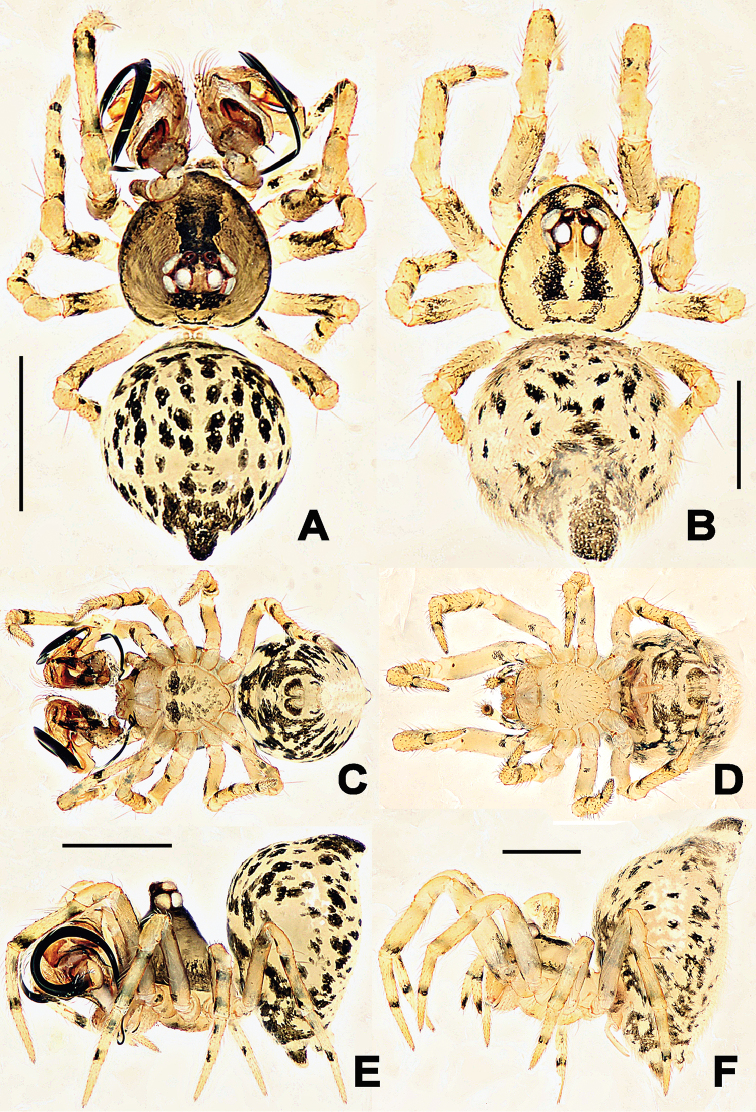
*Troglonetayuensis* Lin & Li, 2013, male habitus (**A, C, E**) and female habitus (**B, D, F**). **A, B** dorsal **C–D** ventral **E–F** lateral. Scale bars: 0.50 mm.

**Figure 7.**
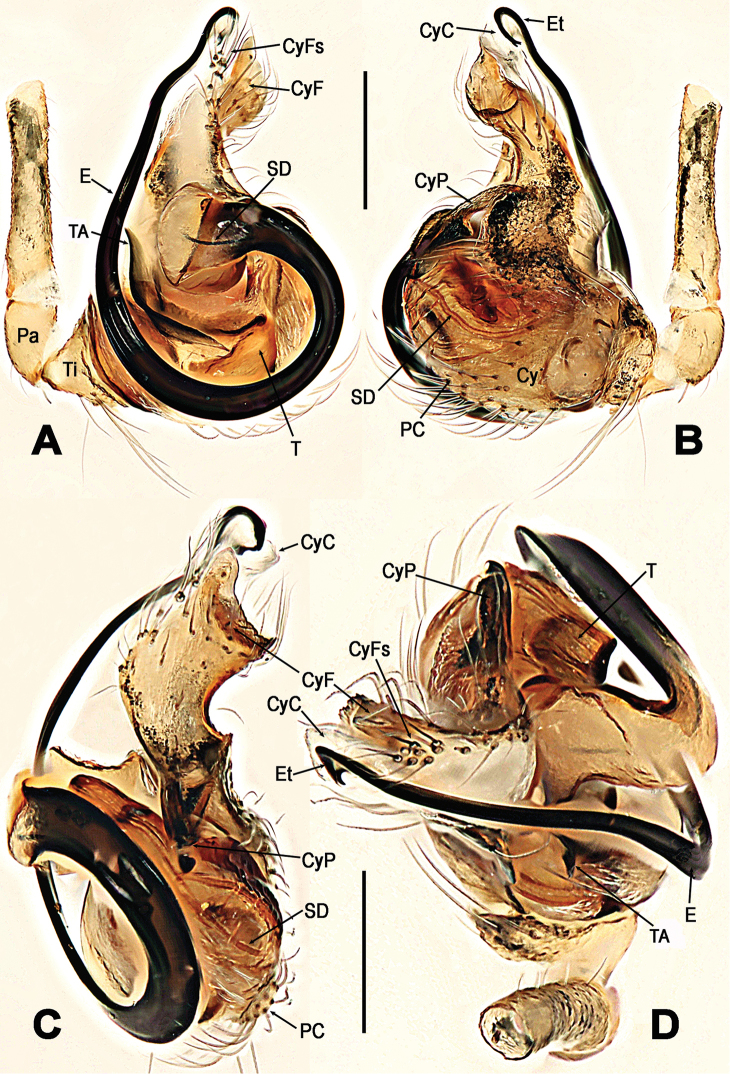
*Troglonetayuensis* Lin & Li, 2013, male left palp (**A–D**). **A** prolateral **B** retrolateral **C** apical **D** ventral. Abbreviations: Cy cymbium; CyC cymbial conductor; CyF cymbial fold; CyFs setae on cymbial fold; CyP cymbial process; E embolus; Et embolic tip; TA tegular apophysis; Pa patella; PC paracymbium; SD spermatic duct; T tegulum; Ti tibia. Scale bars: 0.20 mm.

**Figure 8.**
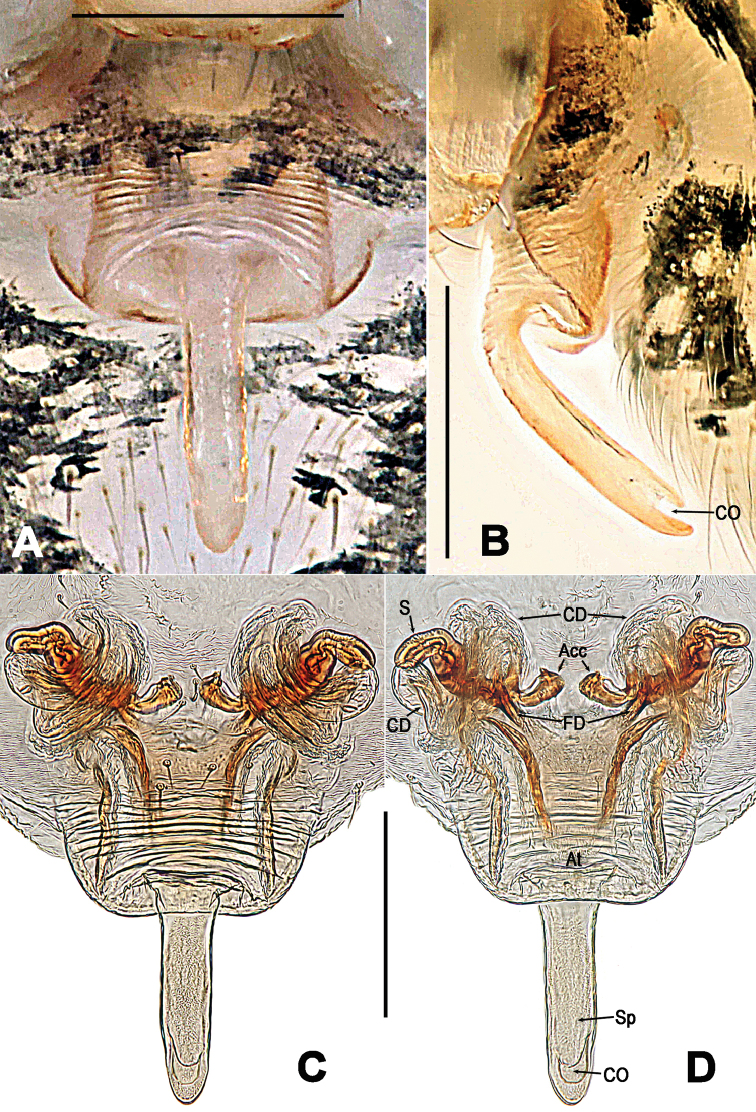
*Troglonetayuensis* Lin & Li, 2013, female epigyne (**A, B**) and vulva (**C, D** lactic acid-treated). **A, C** ventral **B** lateral **D** dorsal. Abbreviations: Acc accessory gland; At atrium; CD copulatory ducts; CO copulatory opening; FD fertilisation ducts; S spermathecae; Sp scape. Scale bars: 0.20 mm.

*Troglonetayuensis* differs from most congeners except the Brazilian *Trogloneta* species and *T.speciosum* by the presence of pointed tubercle on abdomen dorso-posteriorly (Figure 6A, B, E, F). It differs from Brazilian *Trogloneta* spp. (see Brescovit and Lopardo 2008: figs 1D–J, 2C–I, 2K–N) by the presence a large cymbial process of palp and a long epigynal scape (Figs 7A–C, 8A–D); from *T.speciosum* by the larger body size, distinctly stretched cymbial conductor, and long epigynal scape (Figs 7A–C, 8A–D). It is most similar to *T.nojimai* in the male palpal structure and the epigynal configuration, but can be distinguished from the latter by the pointed abdomen dorso-posteriorly, a hooked embolic end, a large cymbial process, and the clavate spermethecae (Figs 7A–C, 8A–D).

#### New morphological data.

**Male. *Measurements***: total length 1.08. Prosoma 0.46 long, 0.45 wide, 0.60 high. Clypeus 0.32 high. Sternum 0.31 long, 0.30 wide. Opisthosoma 0.57 long, 0.55 wide, 0.98 high. Length of legs: I 1.45 (0.44, 0.17, 0.33, 0.29, 0.22); II 1.17 (0.39, 0.16, 0.23, 0.23, 0.16); III 1.00 (0.30, 0.13, 0.22, 0.18, 0.17); IV 1.18 (0.37, 0.15, 0.26, 0.22, 0.18). Legs formula: I-II-IV-III.

***Somatic characters*** (Figure 6A, C, E). *Coloration*: carapace black centrally and marginally, thoracic region dim yellow. Clypeus black. Sternum pale yellow, with a pair of shoulder dark speckles. Abdomen pale yellow, modified by irregular dark spots. *Prosoma*: carapace sub-round dorsally, conical laterally. Cephalic pars sharply raised, slope forward and backward. Ocular area at apex. AME black, others white. AMEsmallest, ALE largest. ALE>PLE>PME>AME. ARE and PRE distinctly procurved. Chelicerae pale yellow, shorter than endites, fang furrow with two promarginal and a single retromarginal teeth. Labium pale, triangular, no fused to sternum. Sternum cordiform, truncated posteriorly. ***Legs***: each segments pale yellow, except tarsi, other segments modified by grey rings distally. A subdistal sclerotized femoral spot on leg I ventrally, but none on leg II. A clasping macroseta on metatarsus I submesial prolaterally. A dorsal seta on each patella distally and tibia proximally. Three trichobothria on tibiae I, II, and IV respectively, but four on tibia III. All metatarsi and tarsi lack trichobothrium. ***Opisthosoma***: elliptic dorsally, triangular laterally, with a tubercle dorso-posteriorly. Spinnerets at lowest position, the anterior ones black, and the posterior ones pale. Colulus small, tongue-shaped. Anal tubercle pale. ***Palp*** (Figure 7A–D): larger than half carapace, strongly sclerotized. Femur 2.5× as long as patella. Patella short. Tibia wider than long, conical, bears a dorsal trichobothrium and a few long setae ventro-marginally. Cymbium large, paracymbium flattened, bears dense long setae. A long cymbial process (aquiline distally, constricted proximally) arisen from inner side subdistal margin of cymbium. Cymbial fold distinct, with long setae. Cymbial conductor extended distally, membranous, attaching with a cluster of setae. Tegulum smooth. Spermatic duct long, visible through subtegulum. A long, fingerlike apophysis derives from the junction between tegulum and subtegulum. Embolus long, arched, gradually tapering to form a hooked end. Embolic end with accessory membrane, hidden behind cymbial conductor.

**Female. *Measurements***: Total length 1.32. Prosoma 0.52 long, 0.49 wide, 0.46 high. Clypeus 0.15 high. Sternum 0.34 long, 0.33 wide. Opisthosoma 0.92 long, 0.88 wide, 1.13 high. Length of legs: I 1.64 (0.53, 0.19, 0.36, 0.31, 0.25); II 1.39 (0.44, 0.18, 0.30, 0.25, 0.22); III 1.15 (0.35, 0.15, 0.25, 0.21, 0.19); IV 1.32 (0.42, 0.17, 0.28, 0.24, 0.21). ***Somatic characters*** (Figure 6B, D, F) as in male, but larger size and lighter colour than male, ocular area more anterior than in male. ***Epigyne*** (Figure 8A–D): Epigynal area elevated ventrally. A long and narrow scape curving basally, copulatory opening at scape distally (Figure 8B). Epigynal plate transversely rugose (Figure 8A, C). Inner vulval structure peculiar (Figure 8D). Copulatory ducts long and bell-shaped proximally (Figure 8D), most duct areas translucent and coiled around the spermathecae (Figure 8C, D). Atrium broad, subquadrate. Spermathecae clavate, curved. Fertilisation ducts stem from the spermathecae baso-dorsally (Figure 8D). Inside accessory gland connected with the base of spermathecae ventrally (Figure 8C).

#### Remarks.

The species is original described on the basis of only a male specimen from Jinyun Mt. of Chongqing City that was donated by Prof Zhisheng Zhang (Southwest University in Chongqing, China) (Lin and Li 2013a). Since then, we have conducted two supplementary collections in the type locality, and not obtained female samples. During 2018, when Mr Guchun Zhou (Hunan Normal University in Changsha, China) was investigating the spider diversity of Yuelu Mt in Changsha City, a large number of samples of this species were obtained by sieving the surface deciduous layers. He presented us with some material for this study.

#### Distribution.

China (Chongqing, Hunan).

### Trogloneta yunnanense

Taxon classificationAnimaliaAraneaeMysmenidae

(Song & Zhu, 1994) comb. n.

Figures 9 , 10 , 11

Pholcomma yunnanense Song & Zhu, 1994: 38, fig. 4A–C; Song, Zhu & Chen, 1999: 127, fig. 66A–B; Li & Lin, 2016: 320.
Trogloneta denticocleari Lin & Li, 2008: 513, figs 16A–E, 17A–E. **Syn. n.**

#### Type material.

Holotype ♀ (of *Pholcommayunnanense*) (IZCAS) from CHINA: Yunnan Province, Gejiu City, Feixia Cave, 4-IV-1992, by hand, D. Song leg. Examined.

#### Other material.

(Types of *T.denticocleari*): Holotype ♂, and paratypes 6♂, 29♀ (IZCAS) from CHINA: Yunnan Province, Kunming City, Panlong District, Xiaohe Town, Yanzidong Cave (25°11.280'N, 102°48.420'E; T. 9 °C; H. 90%; alt. 2,042 m), 4-IV-2007, Y, Lin and J. Liu leg.; 7♂, 11♀ (IZCAS) from CHINA: Yunnan Province, Yiliang County, Jiuxiang Town, Baiyan Cave (25°09.060'N, 103°24.060'E; T. 12 °C; H. 90%; alt. 1,875 m), 9-IV-2007, Y. Lin and J. Liu leg.; 5♂, 7♀ (IZCAS) from CHINA: Guizhou Province, Dafang County, Yangchangba Town, Longdong Village, Qianxudong Cave (27°05.940'N, 105°40.260'E; T. 15 °C; H. 95%; alt. 1,486±14 m), 17-V-2004, Y. Tong and Y. Lin leg.; 2♂, 8♀ (IZCAS) from CHINA: Guizhou Province, Dafang County, Wen’ge Town, Sanhe Village, Yelaodadong Cave (27°10.920'N, 105°28.260'E; T. 10 °C; H. 90%; alt. 1,438 m), 3-V-2007, Y. Lin and J. Liu leg.; 4♂, 5♀ (IZCAS) from CHINA: Guizhou Province, Panxian County, Zhudong Town, Shilipin Village, Shilidadong Cave (25°37.560'N, 104°45.000'E; T. 13 °C; H. 80%; alt. 1,680 m), 15-IV-2007, Y. Lin and J. Liu leg.

#### Supplementary material.

2♂, 18♀ (NHMSU) from CHINA: Yunnan Province, Chuxiong City, Wuning County, Cat street, Xianren Cave (25°27.931'N, 102°10.437'E; alt. 2,066 m), 18-IV-2010, C. Wang, Z. Zhao and L. Lin leg.; 3♂, 1♀ (NHMSU) from CHINA: Guizhou Province, Zunyi City, Shenxi Town, Longjiang Village, Juzizu, Guanniu Cave (27°36.745'N, 106°58.091'E; alt. 814 m), 20-III-2011, Z. Chen and Z. Zha leg.

#### Diagnosis.

**Figure 9.**
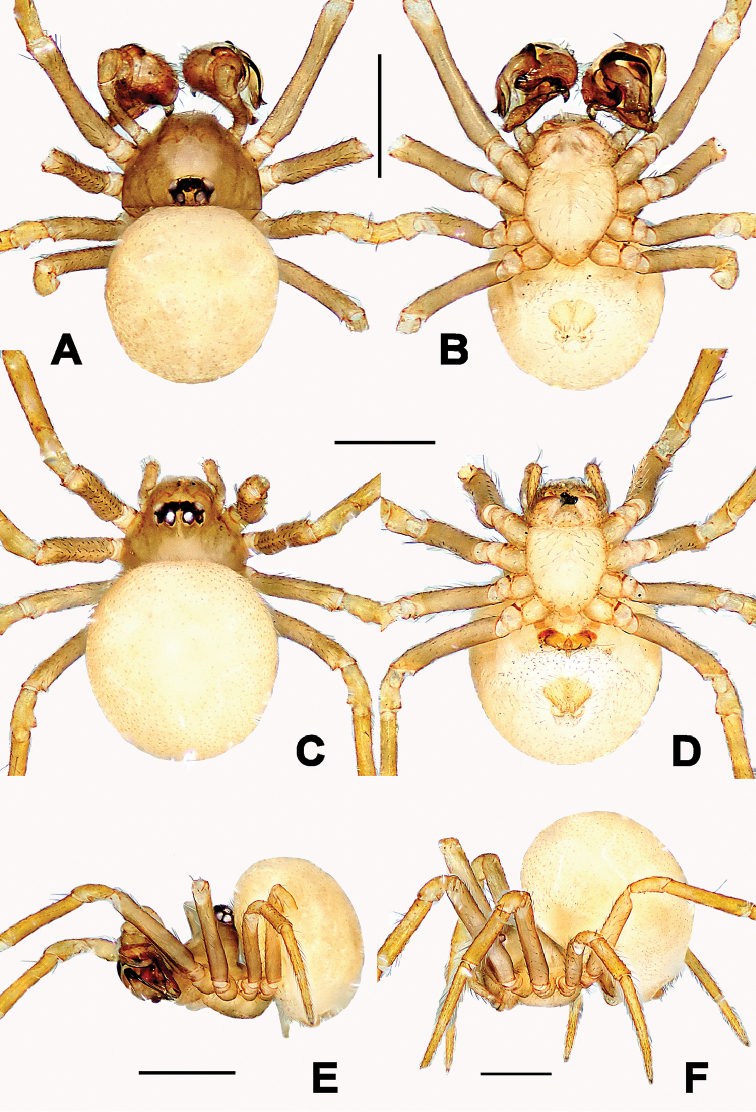
*Troglonetayunnanense* (Song & Zhu, 1994) comb. n., male habitus (**A, B, E**) and female habitus (**C, D, F**). **A, C** dorsal **B, D** ventral **E, F** lateral. Scale bars: 0.50 mm.

**Figure 10.**
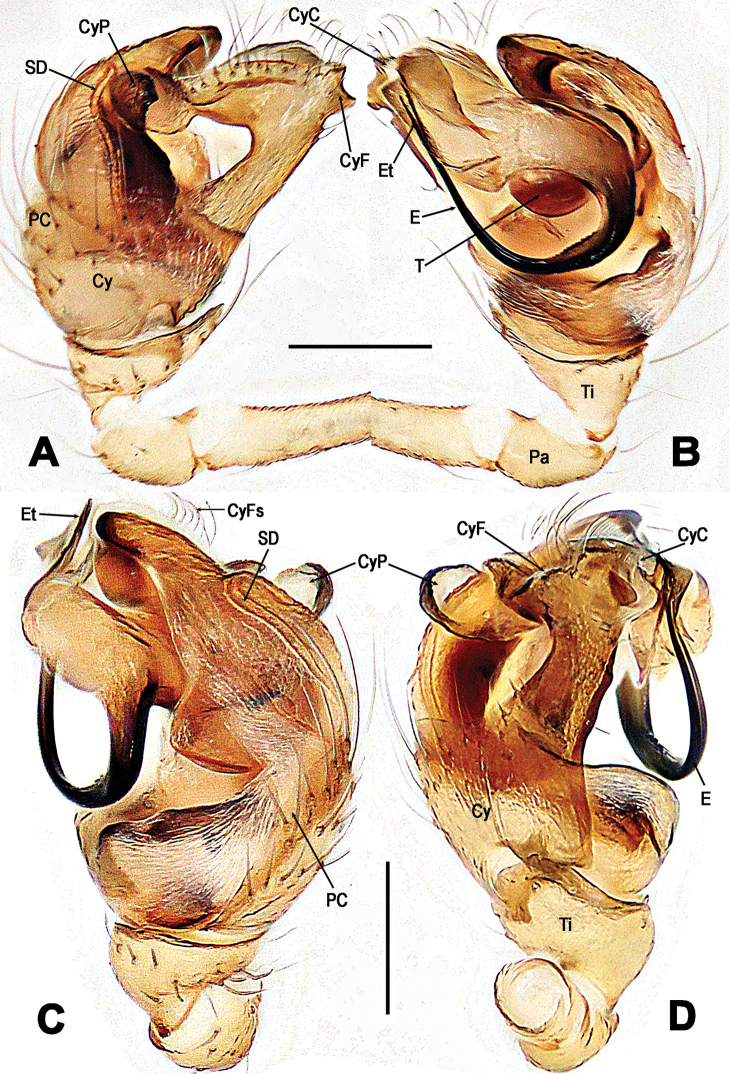
*Troglonetayunnanense* (Song & Zhu, 1994) comb. n., male left palp (**A–D**). **A** prolateral **B** retrolateral **C** dorsal **D** ventral. Abbreviations: Cy cymbium; CyC cymbial conductor; CyF cymbial fold; CyFs setae on cymbial fold; CyP cymbial process; E embolus; Et embolic tip; TA tegular apophysis; Pa patella; PC paracymbium; SD spermatic duct; T tegulum; Ti tibia. Scale bars: 0.20 mm.

**Figure 11.**
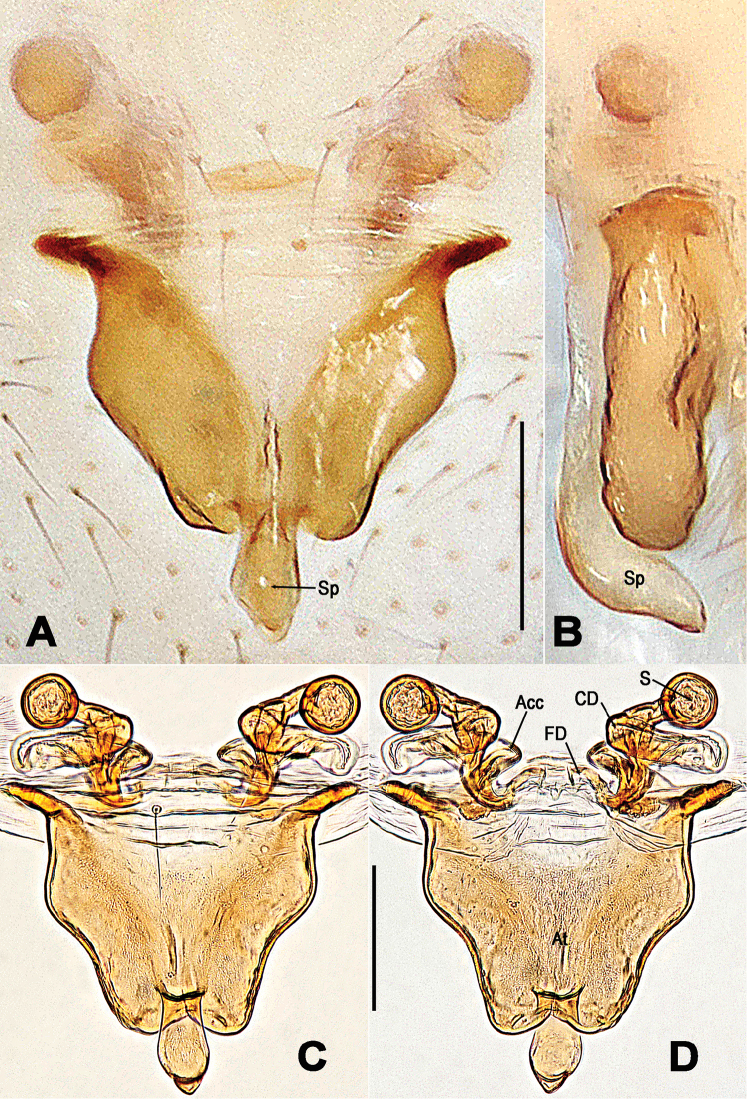
*Troglonetayunnanense* (Song & Zhu, 1994) comb. n., female epigyne (**A, B**) and vulva (**C, D**, lactic acid-treated). **A, C** ventral **B** lateral **D** dorsal. Abbreviations: Acc accessory gland; At atrium; CD copulatory ducts; FD fertilization ducts; S spermathecae; Sp scape. Scale bars: 0.20 mm.

*Troglonetayunnanense* can be distinguished from those *Trogloneta* species with a pointed abdominal tubercle (*T.cantareira*, *T.cariacica*, and *T.mourai* in Brescovit and Lopardo 2008: figs 1A–C, 2A, B, and 2J; and *T.speciosum* in Figure 2A, B, F, G and *T.yuensis* in Figure 6A, B, E, F) by the globular abdomen (Figure 9A–F). It differs from *T.canariensis* and *T.madeirensis* (see Wunderlich 1987: figs 375–380, 382–387) by the long embolus, huge cymbial process, a recurved scape, and the unique broad epigynal plate (Figure 10A, B, 11A, B). It differs from the type species *T.granulum* (see Lopardo and Hormiga 2015: figs 66A–E, 67A, 128F, 131E) and *T.paradoxa* (see Gertsch 1960: figs 12, 15, 16) by a well-developed, spoon-shaped cymbial process and a protruded, pentagonal epigynal plate (Figs 10A, C, D, 11A, B). It differs from *T.uncata* in Figure 5A–D by the long embolus with a straight, tapering distal end (Figure 10B vs. 5A, C) and a huge cymbial process (Figure 10A vs. 5A, D).

#### Taxonomic justification.

The type material of *Pholcommayunnanense* has been examined as well as its related literatures in this study, the habitus features, the body size, the shape of protruded epigyne with an inflexed space, the broad epigynal plate, the configuration of vulva with a pair of far apart, globular spermathecae depicted in the type and original illustrations (Song and Zhu 1994: figs 4A–C) leave little doubt that the identification is correct. The original illustrations of epigyne and vulva of *Pholcommayunnanense* by Song and Zhu (1994) are rather simple and show some differences in comparison with those of *Troglonetadenticocleari* (Lin & Li, 2008: fig. 17A, D–E), but we have examined the type of *Pholcommayunnanense* and the plentiful specimens of *Troglonetadenticocleari*. Therefore, we consider these subtle differences as being intraspecific variation. To further confirm our judgments using DNA barcoding, a survey at the type locality was specially conducted in August 2018. Unfortunately, the habitat of type locality had become so dry that no samples were obtained. Nevertheless, there are valid reasons to think they are synonyms and *T.yunnanense* (Song & Zhu, 1994), comb. n. is proposed and transferred from the genus *Pholcomma* of Theridiidae, as well as *T.denticocleari* proposed as a new synonym.

#### Distribution.

China (Guizhou, Yunnan).

## Discussion

After this study, the genus *Trogloneta* contains twelve nominal mysmenid species. Among its members, three species live in caves (*T.yunnanense*, *T.granulum*, and *T.uncata*), and the other nine are found in surface leaf litter (*T.canariensis*, *T.cantareira*, *T.cariacica*, *T.madeirensis*, *T.mourai*, *T.nojimai*, *T.paradoxa*, and *T.yuensis*) or in forest canopy (*T.speciosum*). Although the genus *Trogloneta* is widely distributed in Europe, Asia, North to South America, and in parts of the Atlantic islands, its many members are clearly endemic species according to the original literature. However, there are two exceptions, and they are *T.granulum* and *T.yunnanense*. The former as the type species of this genus was first found in caves of France, and later reported to be widespread on the surface Beech forest floor and in caves of many European countries, such as Austria, Czech Republic, France, Germany, Italy, Poland, and Slovakia. The latter as a new combination proposed in the current paper is also widely distributed in the isolated limestone caves in Southwest China. So far it has not yet been found on the surface. We know that the caves are relatively closed and isolated habitats: why they have such a distribution pattern, how do they get into caves to eventually colonise them, how does the isolation mechanism of the population work, and other questions are worth further study.

## Supplementary Material

XML Treatment for Trogloneta

XML Treatment for Trogloneta nojimai

XML Treatment for Trogloneta speciosum

XML Treatment for Trogloneta uncata

XML Treatment for Trogloneta yuensis

XML Treatment for Trogloneta yunnanense
